# Anti‐triangle centrality‐based community detection in complex networks

**DOI:** 10.1049/iet-syb.2013.0039

**Published:** 2014-06-01

**Authors:** Songwei Jia, Lin Gao, Yong Gao, Haiyang Wang

**Affiliations:** ^1^ School of Computer Science and Technology, Xidian University Xi'an 710071 People's Republic of China; ^2^ Department of Computer Science University of British Columbia Okanagan Kelowna, British Columbia, Canada V1V 1V7 Canada

**Keywords:** biology computing, complex networks, graph theory, social sciences computing, antitriangle centrality‐based community detection, complex networks, technological networks, social networks, biological networks, vertex properties, edge roles, community discovery, antitriangle property, community structure, edge antitriangle centrality, isolated vertex handling strategy, EACH, antitriangle centrality scores, synthetic networks, real world networks

## Abstract

Community detection has been extensively studied in the past decades largely because of the fact that community exists in various networks such as technological, social and biological networks. Most of the available algorithms, however, only focus on the properties of the vertices, ignoring the roles of the edges. To explore the roles of the edges in the networks for community discovery, the authors introduce the novel edge centrality based on its antitriangle property. To investigate how the edge centrality characterises the community structure, they develop an approach based on the edge antitriangle centrality with the isolated vertex handling strategy (EACH) for community detection. EACH first calculates the edge antitriangle centrality scores for all the edges of a given network and removes the edge with the highest score per iteration until the scores of the remaining edges are all zero. Furthermore, EACH is characterised by being free of the parameters and independent of any additional measures to determine the community structure. To demonstrate the effectiveness of EACH, they compare it with the state‐of‐the art algorithms on both the synthetic networks and the real world networks. The experimental results show that EACH is more accurate and has lower complexity in terms of community discovery and especially it can gain quite inherent and consistent communities with a maximal diameter of four jumps.

## 1 Introduction

The graph or the network is a powerful tool to characterise the complex relations between a set of instances by taking each instance as a vertex and the interaction between a pair of vertices as an edge. Many complex systems can be modelled and analysed as complex networks such as technological networks [1], social networks [2, 3] and biological networks [4, 5] and so on. It has been proved that many real world networks reveal the structures of the modules or the communities that are subgraphs with more edges connecting the vertices of the same group and comparatively fewer links joining the outside vertices. The Modules or the communities reflect the topological relations between the elements of the underlying system and the functional entities. For example, the genes belonging to the same group are prone to reveal a homogeneous biological function; the people in the same social group have the same or similar background or hobbies. Thus, accurately extracting communities has considerable merits in practice because it allows us to infer the special and the hidden relations among the vertices.

However, designing an efficient algorithm for identifying the communities in complex networks is still highly non‐trivial for many reasons. Even though it is non‐trivial, there are several algorithms available. The most popular algorithms maximising the modularity function [6, 7] are criticised for the serious resolution limit problem [8]. The proposed modularity density function solves the resolution limit problem very well [9], however it still is an additional measure to determine the community structure. The methods based on non‐negative matrix factorisation (NMF) [10, 11] and spectral clustering (SC) [12, 13] possess matrix theory supports, but they both depend on a set of parameters. Among these parameters, the number of the expected communities is most important since its determination has direct effectiveness on the results for the real world networks. For more other algorithms for community detection the reader can refer to the literature [14]. Among the algorithms, the centrality algorithms can make use of both the vertex and the edge information. Centrality can be thought of as an important measure to weigh the vertices or the edges in the complex networks. The more important a vertex or an edge is, the larger the centrality is. The essence of these approaches is to discriminate the different roles of the vertices or the edges. For the sake of convenience, the edges connecting various communities are outer links and the inner links are for the same community.

As one of the most famous centralities, edge betweenness [5, 15] is meant to compute the shortest paths between all the pairs of the vertices in a network, and defined as the number of the shortest paths between all the pairs of the vertices through the given edge. However, the GN [5, 15] algorithm based on the edge betweenness is criticised for two reasons: (i) computing the shortest paths between a pair of vertices is expensive; and (ii) the edge betweenness is sensitive to the perturbation of the networks. Furthermore, an edge clustering coefficient [16] is proposed, which is defined as the ratio of the number of the triangles to which a given edge belongs divided by the number of the triangles that might potentially include it. The edge clustering coefficient can decrease the complexity dramatically by sacrificing the accuracy. There are also several other centralities, including information centrality [17], closeness centrality [18], *k* ‐path centrality [19] and so on.

However, none of them can make a good balance between the complexity and the accuracy. This is the major motivation of this paper. We introduce a novel local edge centrality called edge antitriangle centrality for community detection. EACH can be used for large networks since it is just based on the local edge antitriangle centrality. It is characterised by being free of the parameters and independent of any prior measures to determine the community structure. To completely investigate the performance of the proposed centrality, we execute it in comparisons from different aspects: (i) we show the correlation between the edge antitriangle centrality and the edge betweenness, and the anticorrelation between the edge antitriangle centrality and the edge clustering coefficient; (ii) we compare the edge betweenness, the edge clustering coefficient as well as the proposed centrality on the accuracy of characterising the roles of the edges; and (3) we compare the edge antitriangle centrality with the isolated vertex handling strategy (EACH) with the algorithm Girvan and Newman proposed (GN), the algorithm based on the edge clustering coefficient (ECCA) [16], NMF, SC, the algorithm Clauset, Newman and Moore proposed (CNM) [6] and the alogorithm based on spectral maximising modularity density SpeMD [20] on both the synthetic and the real world networks.

The paper is organised as follows: Section 2 introduces the edge antitriangle centrality, Section 3 presents the details of the EACH algorithm, Section 4 shows the experimental results and the conclusions and discussions are proposed in Section 5.

## 2 Edge antitriangle centrality

**Fig. 1:**
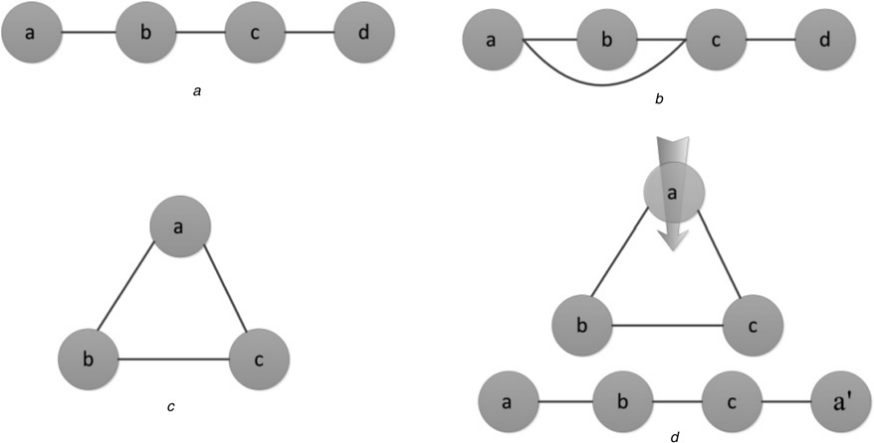
Examples of P_4_, the potential P_4_, the triangle and the antitriangle property of P_4_ *a* P_4_ *a* −*b* −*c* −d *b* Potential *P*_4_ *a* −*b* −*c* −*d* *c* Triangle Δ*abc* *d* Antitriangle property of *P*_4_

Prior to defining the edge antitriangle centrality, we introduce some terminologies that are used in the forthcoming sections. The first is *P*_4_ [21], the second the potential *P*_4_ and the third the triangle. A simple path consisting of four vertices and three consecutive edges is defined as *P*_4_ shown in Fig. 1 *a* and most importantly there is no circle among the four vertices, whereas as shown in Fig. 1 *b* the potential *P*_4_ is not necessarily simple, in other words, the potential *P*_4_ also consists of four vertices and three consecutive edges but there may be circles among the four vertices. What we need to emphasise finally is that the potential *P*_4_ shown in Fig. 1 *b* is not unique and it is just an example of the potential *P*_4_. According to their definitions, *P*_4_ must be the potential *P*_4_, not vice‐versa. A triangle as shown in Fig. 1 *c* consists of three vertices and three consecutive edges, therefore it is the simplest and most basic circle in the complex networks.

The edge antitriangle centrality is defined as the ratio of the number of *P*_4_ to which a given edge belongs divided by the number of the potential *P*_4_ that might include it. The definition is proposed based on the fact that the inner links belong to the more potential *P*_4_ but fewer *P*_4_, whereas the outer links belong to the fewer potential *P*_4_ but more *P*_4_. The denser the edges are, the more circles they belong to. The Intracommunity edges are denser than the intercommunity ones in the complex networks and then there are more triangles including the inner links than the outer links since the triangle is the simplest circle. An edge, for example, *e_ij_*, has more opportunities to be included by the triangles which means it tends to be included by fewer *P*_4_ under the certain degrees of its vertices *i* and *j*. Hence, we can regard *P*_4_ with the property of the antitriangle as shown in Fig. 1 *d*. Thus, there are more *P*_4_ including the outer links than the inner links. There are more potential *P*_4_ including the inner links than the outer links since a triangle is a potential *P*_4_ according to their definitions. Intuitively, we have the fact that the inner links belong to the more potential *P*_4_ but fewer *P*_4_, whereas the outer links belong to the fewer potential *P*_4_ but more *P*_4_.

The edge antitriangle centrality can be used for discriminating the outer links from the inner links for community detection. According to the definition of the edge antitriangle centrality, it can be used to measure the edges to the extent that they can be the inner links and to the extent that they can be the outer links since the larger score an edge has, the more likely it is an outer link, and the lower score an edge has, the more likely it is an inner link.

The antitriangle centrality contains two elements: the number of *P*_4_ and the number of the potential *P*_4_. Given an edge *e_ij_*, the centrality is

(1) $$C_{ij}=\frac{PN_{ij}}{PPN_{ij}}$$

where *PN_ij_* is the number of *P*_4_ and *PPN_ij_* is the number of the potential *P*_4_. To get rid of the degeneracy, we slightly modify the centrality as

(2) $${\bar{C}}_{ij}=\frac{PN_{ij}}{PPN_{ij}+1}$$

To facilitate calculation, we denote the three consecutive edges of the potential *P*_4_ as the left, the central and the right edge, respectively. Correspondingly, we consider the three cases within which a given edge occupies the left, the central and the right position of the potential *P*_4_, respectively, when we calculate *PPN_ij_* and *PN_ij_*.

Let us consider the left, the central and the right case successively and let $PPN_{ij}^{l}$, $PPN_{ij}^{c}$ and $PPN_{ij}^{r}$, respectively, be the number of the potential *P*_4_ with *e_ij_* as its left, central and right edge in sequence. Similarly, the counterparts for *P*_4_ are denoted by $PN_{ij}^{l}$, $PN_{ij}^{c}$ and $PN_{ij}^{r}$, respectively. $PPN_{ij}^{l}$, $PPN_{ij}^{c}$ and $PPN_{ij}^{r}$ can be defined, respectively, as

(3) $$PPN_{ij}^{l}=\sum_{n=1,2,\ldots ,\left|NS(j)\right|.}\left(k_{l_{n}}-1\right),\;l_{n}\in NS(j)$$

(4) $$PPN_{ij}^{c}=(k_{i}-1)\times (k_{j}-1)$$

(5) $$PPN_{ij}^{r}=\sum_{n=1,2,\ldots ,\left|NS(i)\right|}\left(k_{l_{n}}-1\right),\;l_{n}\in NS(i)$$

where *NS* (*j*) is the direct neighbourhood of *j* minus *i*, *NS* (*i*) is the direct neighbourhood of *i* minus *j*, *l_n_* is an arbitrary vertex of *NS* (*j*) or *NS* (*i*) and $k_{l_{n}}$ denotes the degree of *l_n_*. The essence of the calculations of $PN_{ij}^{l}$, $PN_{ij}^{c}$ and $PN_{ij}^{r}$ is to distinguish *P*_4_ from the potential *P*_4_, respectively.

Intuitively, we have

(6) $$PPN_{ij}=PPN_{ij}^{l}+PPN_{ij}^{c}+PPN_{ij}^{r}$$

(7) $$PN_{ij}=PN_{ij}^{l}+PN_{ij}^{c}+PN_{ij}^{r}$$

**Fig. 2:**
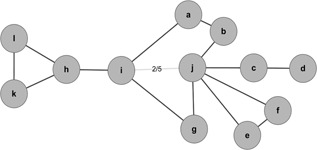
Typical example for computing ${\bar{C}}_{ij}$

Fig. 2 is a typical example for computing the centrality. As shown in Fig. 2, we have *PPN_ij_* = 24 according to (6), *PN_ij_* = 10 according to (7) and ${\bar{C}}_{ij}=2/5$ according to (2), respectively.

## 3 EACH for community detection

### 3.1 EACH and complexity analysis

Without loss of generality, we only consider the connected, the undirected and the unweighted networks, denoted by *G* = (*V*, *E*) where *V* is the set containing all the vertices of the graph *G* and *E* is the set containing all the edges. EACH keeps on removing the edge with the highest edge antitriangle centrality score per iteration until the scores of the remaining edges are all zero. The pseudocode of EACH is described as follows:

**Input** : *G* = (*V*, *E*)

**Output** : *the result communities*

*Calculate the antitriangle centrality score for each available edge*

**While** *the highest score ≠* 0 **do**

*Remove the edge with the highest score*

*Recalculate the scores of those edges affected by the removal*

**End**

*Implement the isolated vertex handling strategy*

*Output the vertices inside the non‐trivial components as those of the result communities*

Let us now analyse the complexity of EACH. First, we focus on the space complexity. The network *G* = (*V*, *E*) with the |*V* | = *N* vertices and the |*E* | = *M* edges can be stored as an *M* × 2 matrix. The edge antitriangle centrality of the *M* edges can be stored as an *M* × 1 matrix. Hence, the total space complexity of EACH is *O* (*M*).

Second, the time computational complexity of the edge antitriangle centrality of *e_ij_*, is

$$O\left(k_{i}k_{j}+\sum_{n=1,2,\ldots ,\left|NS(i)\right|}k_{l_{n}}+\sum_{n=1,2,\ldots ,\left|NS(j)\right|}k_{l_{n}}\right)$$

then $O({\bar{k}}^{2})$ for simplicity, where $\bar{k}$ is the average degree of the network *G*. At the first step of EACH, we calculate the scores of the *M* edges and hence the cost is $O({\bar{k}}^{2}M)$. Then, we calculate the scores of those edges affected per iteration for *T* times since *T* is the maximum number of the iterations and hence the cost is $O({\bar{k}}^{4}T)$. Hence, the whole time complexity of EACH is $O({\bar{k}}^{2}M+{\bar{k}}^{4}T)$ the complexity of the isolated vertex handling strategy can be neglected since there are few isolated vertices in general. On the sparse networks with a very low average degree, EACH is more efficient than others. The space and the time complexities of the other state‐of‐the art algorithms are listed in Table 1, where *K* is the number of the communities and *T*_1_ is the iteration number for searching the parameter for the complexity of the SC, where *d* is the depth of the hierarchy.

**Table 1:** Space and time complexities of the algorithms used in the experiments

Algorithm	Space complexity	Time complexity
GN	*O* (*M*)	*O* (*M* ^2^ *N*)
EACH	*O* (*M*)	$O({\bar{k}}^{2}M+{\bar{k}}^{4}T)$
ECCA	*O* (*M*)	$O({\bar{k}}^{3}M)$
NMF	*O* (*N* ^2^)	*O* (*TKN* ^2^)
SC	*O* (*N* ^2^)	*O* (*MKT* + *NK* ^2^ *T* + *K* ^3^ *T* + *NK* ^2^ *K* ^2^ *T*_1_)
CNM	*O* (*N* ^2^)	*O* (*Md* log*N*)
SpeMD	*O* (*N* ^2^)	*O* (*TN* ^2^)

### 3.2 Details of EACH

EACH keeps on removing until the edge antitriangle centrality scores of the remaining edges are all zero and it may lead to the isolated vertices. What we want to emphasise is that EACH does not need to fix the prior number of the expected communities just because it keeps on removing until the edge antitriangle centrality scores of the available edges are all zero. In fact, the edge antitriangle centrality scores of the available edges are all zero is an additional measure to decide the community structure. In other words, the edge antitriangle centrality possesses the decision role during the edge removing process. For this reason, it does not need to fix the prior number of the expected communities for EACH. To solve the isolated vertices, we handle them by taking advantage of a very simple isolated vertex handling strategy.

Let *N_v_* be the direct neighbourhood of the arbitrary isolated vertex *v* and *V*_NC_ be the set containing all the vertices of the non‐trivial component NC. Then, we define the ratio (|*N_v_* ∩ *V*_NC_ |*/* |*V*_NC_ |) as the measure to [22] quantify the closeness between *v* and NC, where |*N_v_* ∩ *V*_NC_ | is the number of the vertices in the NC connected with *v* and |*V*_NC_ | is the number of the vertices in the NC. If the closeness between *v* and NC is larger than that between *v* and the other non‐trivial components, we select the NC as the candidate component of *v*.

In addition, we solely recalculate the edge antitriangle centrality scores of the few edges in each iteration. For instance, after removing *e_ij_* we just need to recalculate the scores of the edges whose at least one endpoint is belonging to the vertex set *N_i_* ∪ *N_j_*.

## 4 Experiments and analyses

We choose some widely used algorithms including GN, ECCA, NMF, SC, CMN and SpeMD to make comparisons with EACH. The reason why the GN and the ECCA are selected is because they are edge centrality‐based algorithms. The NMF and the SC are based on the matrix theory and the CNM and the SpeMD are based on optimising the additional measures to obtain the expected communities. To completely compare the proposed centrality, we have three types of experiments: first we investigate the relations among the edge betweenness, the edge clustering coefficient and the proposed centrality; then, we compare the three centralities on the accuracy of characterising the roles of the edges; finally, the comparisons are based on community discovery. For convenience, we first list the details of the networks used in the experiments in Table 2 such as the LFR synthetic networks (SNs) [23], the Zachary karate club network (ZKCN) [24], the political blog network (PBN) [25] and the gene regulatory network (GRN) [26], the bottlenose dolphins network (BDN) [27] and the football network (FN) [5, 28], respectively. The parameters of the LFR synthetic network are: average degree $\bar{k}=15$, mixing parameter *mu* = 0.5, minimum for the community sizes minc = 20 and the maximum for the community sizes maxc = 50. Here, we set *mu* = 0.5 because its median is 0.5. In fact, except *mu*, the other parameters are all the defaults of an example inside the original code (http://www.santo.fortunato.googlepages.com/inthe press2).

**Table 2:** Details of the networks used in the experiments

Network	Number of the vertices	Number of the edges	Real number of the communities
SN	1000	7787	32
ZKCN	34	78	2
PBN	1490	16 715	2
GRN	1989	9175	—
BDN	62	159	2
FN	115	613	12

To quantify the accuracy of the algorithms on community discovery, we adopt three widely used criteria: the normalised mutual information denoted as NMI [29], the modularity function denoted as *Q* value [15] and the partition density denoted as the *D* value [30], respectively.

Given two partitions *p*_1_ and *p*_2_ of a network, let ***A*** be the confusion matrix whose element *A_ij_* is the number of the vertices inside the community *i* of the partition *p*_1_ that are also inside the community *j* of the partition *p*_2_. The NMI value *I* (*p*_1_, *p*_2_) is defined as

$$I(p_{1},p_{2})=\frac{-2\sum_{i=1}^{n_{\;p_{1}}}\sum_{\;j=1}^{n_{\;p_{2}}}A_{ij}\log(A_{ij}N/A_{i\cdot }A_{\cdot j})}{\sum_{i=1}^{n_{\;p_{1}}}A_{i\cdot }\log(A_{i\cdot }/N)+\sum_{\;j=1}^{n_{\;p_{2}}}A_{\cdot j}\log(A_{\cdot j}/N)}$$

where $n_{\;p_{1}}\;(n_{\;p_{2}})$ is the number of the communities in the partition *p*_1_ (*p*_2_), *A_i·_* (*A_·j_*) is the sum of the elements of ***A*** in row *i* (column *j*), and *N* is the number of the vertices. A larger value of NMI represents a greater similarity between *p*_1_ and *p*_2_.

The modularity [15] is defined as

$$Q=\sum_{i=1}^{K}\left(\frac{l_{i}}{M}-{\left(\frac{d_{i}}{M}\right)}^{2}\right)$$

where *K* is the number of the communities, *l_i_* is the total number of the edges joining the vertices inside the community *i*, *M* is the total number of the edges in the network and *d_i_* is the sum of the degrees of all the vertices inside the community *i*.

A partition density is used to measure the community structure from the point of view of the edge partitions and does not reveal the resolution limit. For a network with *M* edges, {*p*_1_, …, *p_K_*} is a partition of the edges into *K* communities. Community *p_c_* has *m_c_* = |*p_c_* | edges and $n_{c}=|\cup_{e_{ij}\in p_{c}}\{i,j\}|$ vertices. Then, we have

$$D=\frac{2}{M}\sum_{c}m_{c}\frac{m_{c}-(n_{c}-1)}{\left(n_{c}-2)(n_{c}-1\right)}$$

Obviously, the higher *D* value a partition has, the stronger community structure it possesses.

Testing the networks for community detection consists of ten LFR networks and four practical networks. Here, the GN and the CNM are based on the tool NodeXL (http://www.nodexl.codeplex.com/). The ECCA is implemented by us, the NMF and the SC are based on the R packages NMFN [31] and clusterSim [32], respectively. SpeMD is based on the original code. For the sake of convenience, ECCA_Q indicates the ECCA based on the *Q* value and ECCA_D indicates the ECCA based on the *D* value as additional measures, respectively. EAC indicates the same algorithm as EACH but with no last step of EACH, that is, within the EAC there is no isolated vertex handling strategy. The parameters of the LFR networks are set the same as the synthetic network listed in Table 2 except the mixing parameter there and the mixing parameters here of the ten networks from 0.1 to 1.0 with a step of 0.1. As described in Tables 3, 4, 5–6, we list the *D* value, the *Q* value, the NMI, the edge removal ratio (RR) and the number of the obtained communities (NOC), where there is no NMI in Table 6.

**Table 3:** Results of the GN, the EAC, the EACH, the ECCA_Q, the ECCA_D, the NMF, the SC, the CNM and the SpeMD on the ZKCN

Algorithm	*D*	*Q*	*I*	RR	NOC
GN	0.1656	0.4013	0.5798	100.00%	5
EAC	0.1292	0.3311	0.8048	24.36%	5
EACH	0.1319	0.3715	1.0000	24.36%	2
ECCA_Q	0.1406	0.3245	0.6819	100.00%	5
ECCA_D	0.1435	0.3038	0.5846	100.00%	7
NMF	0.1319	0.3715	1.0000	—	2
SC	0.1319	0.3715	1.0000	—	2
CNM	0.1318	0.3807	0.6925	—	3
SpeMD	0.1319	0.3715	1.0000	—	2

**Table 4:** Results of the GN, the EAC, the EACH, the ECCA_Q, the ECCA_D, the NMF, the SC, the CNM and the SpeMD on the BDN

Algorithm	*D*	*Q*	*I*	RR	NOC
GN	0.1465	0.5194	0.5542	100.00%	5
EAC	0.1945	0.3552	0.3423	54.09%	28
EACH	0.1113	0.4852	0.4434	54.09%	4
ECCA_Q	0.1109	0.3952	0.2354	100.00%	6
ECCA_D	0.1802	0.3694	0.3711	100.00%	14
NMF	0.0947	0.3848	0.8141	—	2
SC	0.0146	0	0.0015	—	2
CNM	0.1261	0.5146	0.5749	—	4
SpeMD	0.0947	0.3848	0.8141	—	2

**Table 5:** Results of the GN, the EAC, the EACH, the ECCA_Q, the ECCA_D, the NMF, the SC, the CNM and the SpeMD on the FN

Algorithm	*D*	*Q*	*I*	RR	NOC
GN	0.3778	0.5950	0.8305	100.00%	8
EAC	0.4172	0.4551	0.8632	50.16%	30
EACH	0.4805	0.5908	0.9113	50.16%	11
ECCA_Q	0.5150	0.6010	0.9065	100.00%	11
ECCA_D	0.5466	0.5805	0.9111	100.00%	13
NMF	0.3940	0.5168	0.8674	—	12
SC	0.4281	0.5516	0.8703	—	12
CNM	0.2728	0.5577	0.7696	—	6
SpeMD	0.5361	0.5959	0.9832	—	12

**Table 6:** Results of the GN, the EAC, the EACH, the ECCA_Q, the ECCA_D, the NMF, the SC, the CNM and the SpeMD on the GRN

Algorithm	*D*	*Q*	RR	NOC
GN	0.0962	0.7604	100.00%	71
EAC	0.1868	0.5676	37.42%	714
EACH	0.1285	0.7024	37.42%	72
ECCA_Q	0.0854	0.5536	100.00%	68
ECCA_D	0.1197	0.4510	100.00%	232
NMF	0.0298	0.0667	—	71
SC	—	—	—	—
CNM	0.0616	0.7279	—	25
SpeMD	0.1629	0.7033	—	69

### 4.1 Relations with the edge betweenness and the edge clustering coefficient

To explore the relations between the edge antitriangle centrality and the edge betweenness and the edge clustering coefficient, we calculate the correlation coefficients and the corresponding *P* ‐values on the synthetic and the real world networks, respectively, as described in Table 7.

**Table 7:** Pearson correlation coefficients and the corresponding *P* ‐values

Network	PCC_AB_	*P* ‐value_AB_ (one‐tailed)	*P* ‐value_AB_ (two‐tailed)	PCC_AE_	*P* ‐value_AE_ (one‐tailed)	*P* ‐value_AE_ (two‐tailed)
SN	0.6795	0	0	−0.8794	0	0
ZKCN	0.5245	4.100 × 10^−7^	8.300 × 10^−7^	−0.3777	3.2588 × 10^−4^	6.5176 × 10^−4^
PBN	0.3504	0	0	−0.4362	0	0
GRN	0.5536	0	0	−0.3473	0	0

**Fig. 3:**
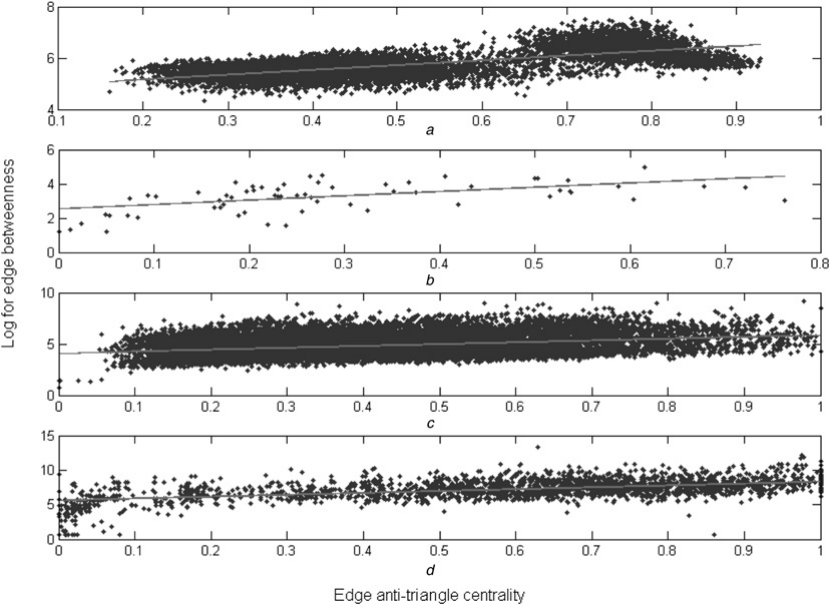
Scatters plots for the edge antitriangle centrality and the logarithm for the edge betweenness, where the red lines are their corresponding curves to fit them *a* On the SN *b* On the ZKCN *c* On the PBN *d* On the GRN

**Fig. 4:**
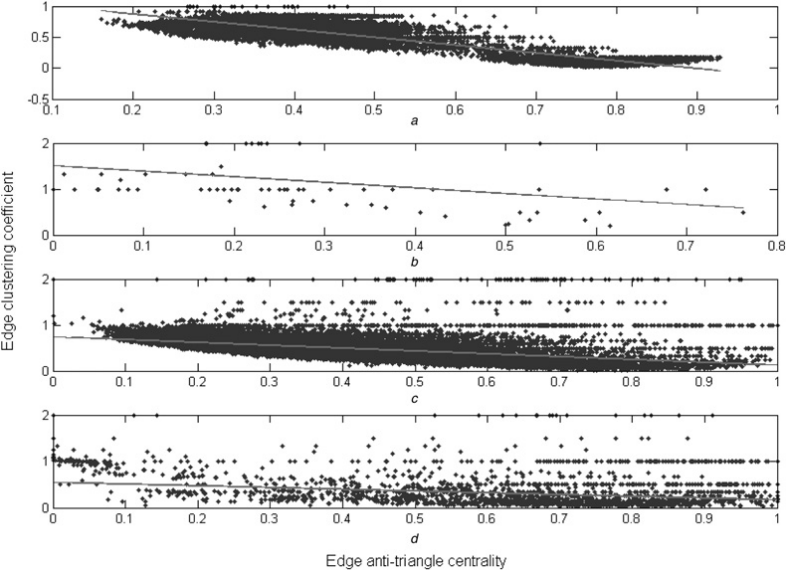
Scatters plots for the edge antitriangle centrality and the edge clustering coefficient, with the same details as Fig. 3 *a* On the SN *b* On the ZKCN *c* On the PBN *d* On the GRN

As shown in Fig. 3 *a*, we plot the scatters of the edge antitriangle centrality and the logarithm of the edge betweenness on the SN, a typical artificial network. The two centralities are positively correlated because the Pearson correlation coefficient is 0.6795 and their two type corresponding *P* ‐values are all zero. This means that the edges with higher edge antitriangle centrality scores tend to have higher edge betweenness. As shown in Fig. 4 *a*, we plot the scatters of the edge antitriangle centrality and the edge clustering coefficient on the same network. Obviously, an anticorrelation between these two centralities for the Pearson correlation coefficient is −0.8794 and their two types corresponding *P* ‐values are also zero. Then, the edges with higher edge antitriangle centrality scores tend to have lower edge clustering coefficient scores.

Following on, Figs. 3 *b* and 4 *b* show their relations on the ZKCN, a typical small social network. Figs. 3 *c* and 4 *c* show them on the PBN, a typical medium social network. Figs. 3 *d* and 4 *d* show them on the GRN, a typical biological network. In fact, Fig. 3 reveals the correlations and Fig. 4 reveals the anticorrelation.

The correlation between the edge antitriangle centrality and the edge betweenness, the anticorrelation between the edge antitriangle centrality and the edge clustering coefficient are inherent on various networks. Thus, the edge antitriangle centrality can be possible for community detection such as edge betweenness and edge clustering coefficient.

### 4.2 Accuracy on characterising the roles of the edges

Here, in order to compare the three centralities on the accuracy of characterising the roles of the edges, we use two important quantities, respectively. The first one is the fraction of the vertices contained in the giant component, denoted by RGC [33]. A sudden decline of the RGC is observed if the network disintegrates after the deletion of a certain fraction of the edges. Another quantity is the so called normalised susceptibility [33], defined as

(8) $$\tilde{S}=\sum_{s<s_{\max}}\frac{n_{s}s^{2}}{N}$$

where *n_s_* is the number of the components with size *s*, *N* is the size of the whole network and the sum runs over all the components except the largest one. When $\tilde{S}$ is a function of the fraction of the removed edges *f*, usually, an obvious peak can be observed that corresponds to the precise point at which the network disintegrates [33, 34]. We compare the three centralities on those networks used in Section 4.1.

**Fig. 5:**
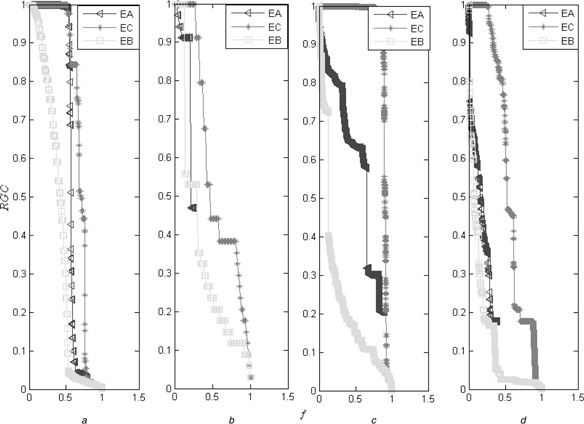
Comparison from the point of view of the RGC, where the edge antitriangle centrality is denoted by EA, the edge clustering coefficient is denoted by EC and the edge betweenness is denoted by EB *a* On the SN *b* On the ZKCN *c* On the PBN *d* On the GRN

**Fig. 6:**
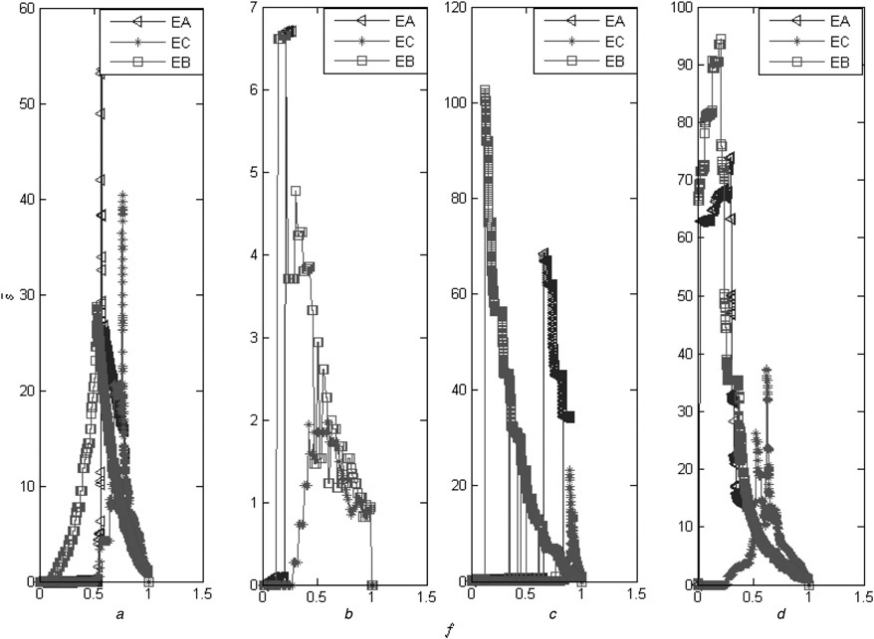
Comparison from the point of view of $\tilde{S}$, with the same details as Fig. 5 *a* On the SN *b* On the ZKCN *c* On the PBN *d* On the GRN

As shown in Fig. 5, we compare the three centralities from the point of view of the RGC. As shown in Fig. 5, the edge antitriangle centrality reveals the comparative accuracy compared with the edge betweenness. However, as shown in Fig. 5, the edge antitriangle centrality reveals more accuracy than the edge clustering coefficient on the four typical networks. As shown in Fig. 6, we compare them from the point of view of the normalised susceptibility. The results also demonstrate that the edge antitriangle centrality reveals the comparative accuracy compared with the edge betweenness which has more accuracy than the edge clustering coefficient.

### 4.3 Community detection results

For the length limit, the analyses of the synthetic networks and the social networks are arranged in the Supplementary Materials. Here, we show the main results of the GRN.

#### 4.3.1 Gene regulatory network

**Fig. 7:**
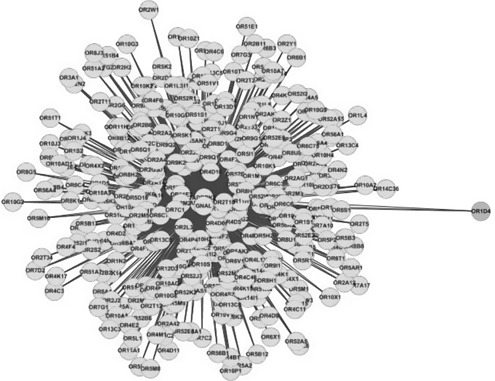
Largest module obtained by EACH, GN, EAC and SpeMD consisting of 353 genes

Through the GRN from the literature [26], we get rid of the genes with no official name and neglect all the directions. A vertex indicates a gene and an edge indicates a regulatory relation between the two genes. As described in Table 6, the *D* value and the *Q* value of EACH are 0.1285 and 0.7024, respectively. The *D* value of EACH is higher than that of the GN, the *Q* value is close to that of the GN. The edge RR is just 37.42% much less than that of the GN. The isolated vertex handing strategy improves the *Q* value from 0.5676 to 0.7024 and the number of the communities (the modules in the biological networks) from 714 to 72 closest to the number obtained by the GN. As shown in Fig. 7, the largest module of the results obtained by EACH, GN, EAC and SpeMD, respectively, is the same one including 353 genes. We make an analysis of these 353 genes by the web tool Gene Trail Express [35]. Fortunately, among these 353 genes there are 352 ones belonging to the subcategory olfactory transduction and the corresponding *P* ‐value is 0. The 352 genes are green as shown in Fig. 7 and only the gene OR1D4 is not a member of the subcategory olfactory transduction. As shown in Fig. s3 (supplementary materials), the largest module of the results obtained by the ECCA_D consists of 410 genes. However, there are only 352 genes (green ones) among these 410 ones belonging to the subcategory olfactory transduction. Obviously, the remaining 58 genes (pink ones) and the 352 genes belong to different modules, but regretfully, the pink ones are not extracted from the largest module by the ECCA_D. As shown in Fig. s4, the largest module of the results obtained by the ECCA_Q consists of 932 genes. However, there are only 352 genes (green ones) among these 932 ones belonging to the subcategory olfactory transduction. Obviously, the remaining 580 genes (pink ones) and the 352 genes belong to different modules, but regretfully, the pink ones are not extracted from the largest module by the ECCA_Q. In addition, intuitively, there are obvious module structures inside the 580 genes but the ECCA_Q cannot detect them further. As for the NMF, especially, we set the prior number of the expected modules as 71 the same as that obtained by the GN, then the largest module of the results obtained by the NMF consists of 123 genes. However, there are no regulatory relations among these genes. The largest module of the results obtained by the CNM consists of 516 genes, however there is no significant biological function among them. Here, as for the SC we also set the prior number of the expected modules as 71 Since the SC runs over 180 h on this network but does not output any results, we stop the R package.

**Fig. 8:**
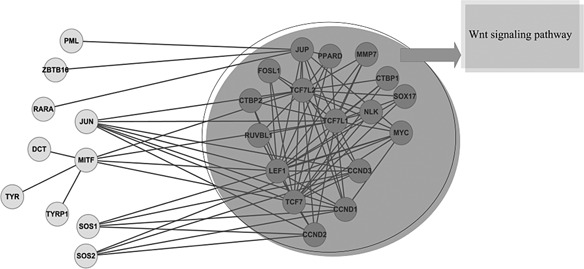
One of the particular modules obtained by EACH consisting of 27 genes and revealing a significant biological function

From the point of view of the whole results, the GN obtains 71 modules and there are 12 modules only including one gene. The EAC obtains 714 modules and there are too many modules only including one gene. The EACH obtains 72 modules, the ECCA_Q obtains 68 modules, the ECCA_D obtains 232 modules, the SpeMD obtains 69 modules and the CNM obtains 25 modules, respectively. By comparing the results obtained by EACH with those of the other algorithms, we can take advantage of the neighborhood affinity score to decide one module when matching the other modules [36]. Among the 72 modules obtained by EACH, there are 53 modules matching and 19 ones not matching those of the GRN, there are 41 ones matching and 31 ones not matching those of the ECCA_Q, there are 64 modules matching and 8 ones not matching those of the ECCA_D, there are 27 modules matching and 45 ones not matching those of the NMF and the CNM and there are 59 modules matching and 13 ones not matching those of the SpeMD, respectively. Then, these common modules reveal the robustness of EACH and the particular ones reveal its novelty. What we want to emphasise is that there are two modules obtained by EACH, which do not match any module obtained by the other algorithms in this paper. One module consists of 115 genes and reveals no significant biological function, whereas the other module consists of 27 genes, further among the genes of this module there are 17 ones belonging to the subcategory Wnt signalling pathway and the *P* ‐value is 9.0 × 10^−22^, as shown in Fig. 8. Hence, in general, EACH can obtain more meaningful and more compact communities in this network.

#### 4.3.2 Advantages of EACH

We can find several advantages of EACH very intuitively by systematic comparisons. Firstly, the performance of the isolated vertex handing strategy within EACH is significant. Secondly, EACH is more accurate than those that do not depend on the prior number of the communities on most networks. Thirdly, unlike the NMF, the SC and the SpeMD, EACH is free of parameters. What we want to emphasise here is that it does not need to fix the prior number of the expected communities and the number can be fixed automatically during the edge removing process. Fourthly, unlike the ECCA, EACH does not depend on any additional measures to decide the community structure and what is more important, it can obtain inherent and consistent communities. Fifthly, the complexity of EACH is significantly lower than others. Finally, the communities obtained by EACH are more compact than others and the diameters of the communities are four jumps at most. Thus, EACH is more appropriate for the networks with compact community structures.

## 5 Conclusions and discussions

In this paper, we propose a novel local edge antitriangle centrality and further propose our approach (EACH) based on this centrality for community detection. EACH is characterised by being free of any parameters including the prior number of the expected communities and independent of any additional measures to decide the community structure. We demonstrate that the novel local edge antitriangle centrality is appropriate for community detection as the edge betweenness and the edge clustering coefficient and we follow up on testing EACH and the other state‐of the‐art algorithms on several synthetic and practical networks, the experimental results show that EACH is more efficient and accurate and especially can gain quite inherent and consistent communities with a maximal diameter of four jumps. Thus, EACH is more appropriate for the networks possessing compact community structures inside themselves.

Although EACH owns outstanding properties, there are still some problems requiring further investigation. Firstly, the isolated vertex handling strategy used in this paper reduces the performance of EACH on the LFR networks when the mixing parameter *mu* ≥ 0.6. As for the LFR networks, there are more isolated vertices left as *mu* increases, while the isolated vertices handling strategy used in this paper cannot handle these isolated vertices very effectively. Therefore seeking a better isolated vertex handling strategy deserves further research. Secondly, the edge antitriangle centrality is designed for the undirected and the unweighted networks. Next we want to extend this centrality for the directed and the weighted networks. Finally, although the edge antitriangle centrality is developed for community detection, we can seek other usages.
